# Antivaccine Messages on Facebook: Preliminary Audit

**DOI:** 10.2196/18878

**Published:** 2020-10-20

**Authors:** Dhamanpreet Dhaliwal, Cynthia Mannion

**Affiliations:** 1 University of Calgary Calgary, AB Canada

**Keywords:** antivaccine, vaccines, vaccination, immunization, communicable disease

## Abstract

**Background:**

The World Health Organization lists vaccine hesitancy as one of 10 threats to global health. The antivaccine movement uses Facebook to promote messages on the alleged dangers and consequences of vaccinating, leading to a reluctance to immunize against preventable communicable diseases.

**Objective:**

We would like to know more about the messages these websites are sharing via social media that can influence readers and consumers. What messages is the public receiving on Facebook about immunization? What content (news articles, testimonials, videos, scientific studies) is being promoted?

**Methods:**

We proposed using a social media audit tool and 3 categorical lists to capture information on websites and posts, respectively. The keywords “vaccine,” “vaccine truth,” and “anti-vax” were entered in the Facebook search bar. A Facebook page was examined if it had between 2500 and 150,000 likes. Data about beliefs, calls to action, and testimonials were recorded from posts and listed under the categories Myths, Truths, and Consequences. Website data were entered in a social media audit template.

**Results:**

Users’ posts reflected fear and vaccine hesitancy resulting from the alleged dangers of immunization featured on the website links. Vaccines were blamed for afflictions such as autism, cancer, and infertility. Mothers shared testimonies on alleged consequences their children suffered due to immunization, which have influenced other parents to not vaccinate their children. Users denied the current measles outbreaks in the United States to be true, retaliating against the government in protests for fabricating news.

**Conclusions:**

Some Facebook messages encourage prevailing myths about the safety and consequences of vaccines and likely contribute to parents’ vaccine hesitancy. Deeply concerning is the mistrust social media has the potential to cast upon the relationship between health care providers and the public. A grasp of common misconceptions can help support health care provider practice.

## Introduction

Many diseases have been almost, or completely, eradicated due to immunization. Immunization against disease prevents 2-3 million deaths per year internationally and could prevent even more with global vaccination improvements [1]. Immunization has vastly decreased mortality due to preventable communicable diseases. For example, before the introduction of the measles vaccine, 300,000-400,000 Canadians were infected every year, with some recoveries and many deaths [2]. Since the elimination of measles in 1998 due to vaccines, there have been very few cases in Canada [2]. Similarly, once the polio vaccine was introduced in Canada in the 1950s, cases reduced dramatically, and the current risk to the Canadian population is extremely low [3].

The World Health Organization (WHO) has declared vaccine hesitancy as one of the top 10 threats to global health [4]. Social media has helped fuel the growth of the antivaccine movement, with Facebook being identified as a key disseminator of misinformation surrounding the campaign [5-7]. Facebook is the largest social media platform, with more than 2 billion active monthly users [8]. There have been serious efforts to reduce the amount of misinformation spread on the social media site by lowering the ranking of Groups and Pages making false claims [7]. Social media administrators have been urged to remove these Pages and Groups altogether; however, counterarguments cite a violation of human rights to access uncensored information [7]. This paper exposes the messages of the antivaccine movement online and how individuals perceive immunization. We aimed to uncover the myths and truths that users of Facebook Pages observe and partake in. Health care consumers and health care providers may find themselves on opposite ends of the debate. Lack of immunization places the public at risk and decreases public health efforts to curb measles and polio and prevent outbreaks of influenza (flu) along with other communicable diseases. The shift in power between doctors and patients due to easy access to information online has led to the questioning of health care providers and increased shared decision making [6,9].

As most of the world awaits a vaccine to put an end to the COVID-19 (also known as the 2019 novel coronavirus) pandemic, “followers” of antivaccine Facebook Pages seem to fear the vaccine more than the virus itself [10]. Amid the COVID-19 pandemic, social media sites such as Facebook are unable to control the health misinformation that is spread on its Pages [11]. Antivaccine Pages have been providing conspiracy theories, safety concerns, and alternative health medication that grasp the attention of “undecided” individuals surfing the web for information on vaccines [11]. The WHO is fighting to stop the spread of misinformation online by collaborating with social media giants to find a way to regulate false claims [12]. Some examples of such claims include that COVID-19 is a bioweapon funded by the Bill & Melinda Gates Foundation or that it can simply be cured by consuming homemade concoctions (some include drinking bleach) [13]. Our aim was to know more about the messages that can influence readers and consumers that these websites are sharing via social media.

## Methods

Publicly available content on 4 Facebook Pages was analyzed based on the number of likes they received. Keywords “anti-vax,” “anti-vaccine,” “vaccine,” “vaccine injury,” and “stop vaccination” were entered into the Facebook search bar. Once on the “results” page, we followed the link to the “Pages” tab. Pages were chosen if they had between 2500 and 150,000 likes — a measure of the spread of readership. This range was selected based on the fact that it included most pages that had high traffic with daily activity. The Page was selected if it had the highest amount of likes on the first “results” page. We then scrolled down to January 1, 2019 and analyzed posts, comments on posts, and website links shared until May 30, 2019. Flu activity peaks between December and February and can last as late as May [14]. Website and posts data simultaneously reached saturation, the point where no new themes emerged. Website links shared on the Facebook Pages were publicly accessible and consisted of news articles, blog posts, scientific studies, or website posts of renowned antivaccine activists. The data collected from the Facebook posts were categorized into “Myths,” “Truths,” and “Consequences.” These lists helped categorize the data found on the Facebook Pages to determine the exaggeration of myths and falsehoods and the minimization of truths. A separate category, “Measles Outbreak Reactions,” was used to document reactions to outbreaks of measles happening around the United States that were garnering attention in mainstream media and on the Facebook Pages.

Website data captured from links shared between January 2019 and May 2019 were entered into the Who, What, When, and Why categories of the social media audit template. Using this tool, we were able to capture and categorize data in a uniform manner for all websites. The social media audit template was created by Keith Quesenberry [15]. He describes a social media audit as “a systematic examination of social media data” [15]. We adapted and modified this tool to help us gain insight on what points and messages website authors are trying to get across. This tool is to be used to systemically examine “social talk of a brand” — in this case, the “brand” is the topic of immunization [16] — and allows the examiner to shift their viewpoint from “control” to “engagement” and understand why users are participating in such forums by examining specific content and posts [16]. The “Who” category captured the type of website (eg, blog post, news article) and the URL, which helped us determine the type of websites that were being shared. The “What” category was used to describe what content the website was sharing. This category was crucial in helping us determine the messages of the website. “When” noted the date the website content was published to determine whether links are being shared on the Facebook Page instantaneously or randomly. In the “Why” category, we noted any comments or statements made by the Facebook Page when sharing the linked website. Noting these statements in this section helped give us a better idea of the purpose behind sharing these websites and what the administrators of the page hoped to achieve by sharing these links with their audience. “Opportunity” was a crucial category in helping us note the amount of “reactions,” “shares,” and “comments” on the Facebook post sharing the link. By noting the reactions, we were able to assert which type of links get the most reactions and replies from the audience.

## Results

### Myths

The claims made by the authors on the Facebook Pages were diverse and ranged from questioning the ethics of administration to a total disregard of the benefits of immunization. Demographics of the overseers of the Facebook Page or users cannot be known as Pages can be accessed worldwide; however, given the posts about the Centers for Disease Control and Prevention (CDC) and current events in the United States, we conjecture that the users and majority of commentators are from the United States. Claims under “Myths” numbered far greater than those listed under “Truths.” Claims are listed in order of greatest to least in number.

#### Claim 1: “Vaccines Fail”

Several users on all Facebook Pages expressed the concern that vaccines are not 100% safe and people should opt to not vaccinate. References were made to the recent outbreaks of measles in the United States, and users claimed that most affected individuals were already vaccinated. The CDC reports that a majority of measles-affected individuals were unvaccinated [17]. Claims were made that the measles/mumps/rubella (MMR) vaccine was failing, and the need for the DTaP vaccine for tetanus was disregarded, as tetanus is not a communicable disease.

#### Claim 2: “Vaccine Schedules are Overwhelming and Spark Autoimmunity”

Users expressed concerns with the number of immunizations being added to child schedules by the CDC and with multiple vaccines given at one time. Parents were concerned about vaccines overstimulating the immune system. Some parents claimed to go with “alternative” immunization schedules in collaboration with their health care providers [18]. This included giving fewer vaccines at once and skipping vaccines deemed “not important” by the parents. Users also expressed concerns about the differences in child vaccine schedules among different countries and used it as a reason not to vaccinate against certain diseases (eg, the United Kingdom does not vaccinate against varicella).

#### Claim 3: “Vaccines Contain Harmful Adjuvants”

Adjuvants used in vaccines have been under heavy scrutiny on all Facebook Pages. Adjuvants are added to a vaccine to strengthen its ability to stimulate the immune system [19]; however, they are believed to be responsible for causing a variety of diseases such as cancer, infertility, Alzheimer’s, and autism. Each vaccine has been linked to its own set of mythical consequences from contained adjuvants (see Table 1). Table 1 lists the most popular vaccines discussed on the Facebook Pages with the most popular adjuvant in each vaccine that is accused of causing harm. The table highlights the effect of the disease on unprotected individuals to reinforce the dangers of the infections prevented by immunization.

**Table 1:** Vaccines and their alleged consequences.

Diseases that can be prevented by immunization	Available vaccine(s)	Adjuvant in vaccine allegedly causing adverse effects	Prevailing myth(s)
Human papillomavirus	Gardasil, Gardasil 9	Polysorbate 80, aluminium	Infertility, premature ovarian failure, paralysis
Measles, mumps, rubella (MMR)	MMR	Aluminum, fetal bovine serum, recombinant human albumin	Autism, seizures, measles shedding from a vaccine, Alzheimer’s, lupus, aseptic meningitis
Diphtheria, pertussis, tetanus	Dtap, Tdap	Formaldehyde, polysorbate 80, bovine serum albumin	SIDS^a^, autism, vaccine-induced pertussis, neurodevelopmental problems, miscarriage, death
Polio	Inactivated poliovirus	Simian virus 40	Cancer, vaccine-induced paralysis

^a^SIDS: sudden infant death syndrome.

The human papillomavirus vaccine was heavily linked to infertility and polycystic ovarian syndrome, MMR vaccine to autism and epilepsy, polio vaccine to cancer, and DTaP vaccine to sudden infant death syndrome (SIDS). Vaccines are accused of containing fetal cells as adjuvants, and claims are made that they influence the sexuality of teenagers and lead to homosexuality.

### Truths

“Truths” contained information shared on Facebook that could be supported by peer-reviewed scientific evidence. Repeated concerns were raised over the efficacy of the flu vaccine. Flu vaccines are in production before the flu season begins (meetings in February for the Northern Hemisphere and in September for the Southern Hemisphere), and this information was shared on the Facebook Pages. Flu strains are predicted based on surveillance, laboratory, and clinical studies [20]. The flu vaccine’s effectiveness was questioned on all Facebook Pages; however, the effectiveness of the flu shot can change every year [21]. The Government of Canada states that the flu virus may change while the vaccine is in production: “Even when there is a less-than-ideal match or lower effectiveness against one virus, the seasonal flu shot can still provide protection against the remaining two or three viruses. If you do get the flu, the flu shot may reduce the severity of your symptoms” [21].

Another concern raised was “over-vaccinating” against pertussis (or whooping cough) as the vaccine is allegedly not effective. Schwartz et al [22] found that 4 years postimmunization, immunity to pertussis declined significantly, especially with the acellular vaccine as compared to the whole-cell vaccine. Booster shots during pregnancy or priming with the whole-cell vaccine are recommended to optimize pertussis control [22]. Use of the acellular vaccine instead of the whole-cell vaccine was another topic discussed on the Facebook Pages. A case-control study by Klein et al [23] found that, among teenagers who had received vaccines for pertussis at Kaiser Permanente Northern California, those immunized with whole-cell vaccine were more protected in outbreaks compared to teenagers who received the acellular pertussis vaccine.

### Consequences

Autism is the most widely known affliction that allegedly implicates vaccines. The most popular consequences linked to vaccines also included SIDS, asthma, epilepsy, cancer, Alzheimer’s, miscarriage, infertility, and death (see Table 1). Testimonies from parents sharing information about the death of their children and posting their pictures are extremely popular on all the Pages. These have a profound effect on other viewers, as evidenced by their responses. Mothers have shared their hesitancy of vaccinating their children after viewing these posts.

### Reactions to Current Measles Outbreaks

Measles outbreaks are at an all-time high in the United States, since the year 1992, with the numbers of cases growing. To achieve herd immunity for measles, 95% of the population must be immune to the infection [24]. Outbreaks in communities that are unvaccinated in New York account for more than 75% of the cases, with the majority affecting Orthodox Jewish communities where immunization rates are low. All Facebook Pages discussed the current coverage of measles outbreaks; however, 2 of the 4 Pages we examined posted more frequently about the mainstream media coverage of the outbreaks. Reactions to outbreaks included denial of events, accusing mainstream media of falsifying reports, and claiming that most individuals spreading the infection were vaccinated. While a vaccinated individual could contract measles, the chances are much lower compared to those for unvaccinated individuals, and the disease presents in a milder form [21].

### Website Data

Website links shared on the Facebook Pages were followed. Data were also collected from January 2019 to May 2019. Website information was categorized into Who, What, When, Why, and Opportunity.

#### “Who”

We found that the shared website links were predominately blog posts coming from antivaccine activists. The most popular website shared on all the Pages was Green Med Info [25]. The author, Sayer Ji, is a self-proclaimed expert on the rights and wrongs of immunization and supports alternative medicine [25]. Other types of shared websites included news article, testimonials, and studies. Robert Kennedy is a huge supporter of antivaccine sentiments. His blog website was shared numerous times on all Pages.

#### “What”

We found that the themes emerging from shared content on the websites varied. These included stories of vaccinated individuals getting the infection they were vaccinated against, testimonies from mothers whose children allegedly died postimmunization, accusations towards the government and physicians for promoting vaccines to make money, condemnation of mandatory vaccine bills and laws, “expert” testimonies on dangers of immunization, promotion of naturopathic medicine, and denial of the harm of illnesses that vaccines protect from. Some websites claimed that childhood infection with measles provides protection from cardiac disease in adult life and women will be protected from ovarian cancer, but the sites do not produce sufficient evidence to support these claims.

#### “When”

Information on the vast majority of the websites had been published within the last month; however, for sites that were not current, they were at least published in the last decade. The growth of these Pages in the last decade supports the fact that antivaccine content has increased with the use of social media and the internet [6,26]. The “post” or “share” date on the Facebook Pages compared to the original publication date of the website was within 1 week for most posts on 3 of the 4 Pages. We found that one Facebook Page in particular shared website links published 1 or 2 years before current events, but still received lots of support from followers.

#### “Why”

The main reasons for sharing a website link were either to promote or condemn its content. The majority of website links were supported and shared as a way of endorsing the antivaccine information. We do not have reason to believe that any of the shared website links were vetted for scientific evidence or truthfulness; instead, content that would have a profound effect on viewers was shared. This included “new” studies and information such as the “benefits” of getting an infection.

#### “Opportunity”

We found that comments, “reactions,” and “shares” on the Facebook posts sharing the website link did not show any specific trend. The number of responses varied heavily; this could be attributed to the number of online users or personal interests. Therefore, we cannot make a conclusion on whether any specific topics sparked user interest; however, a news article shared on one of the Pages about Kailyn Lowry, an actress, had the highest number of “reactions” compared to any other website link shared on one of the Facebook Pages. This is significant, as celebrities have the platform to influence many people across different geographical areas just by sharing their personal beliefs [9,27].

## Discussion

### Principal Findings

The analysis of the Facebook Pages led to emerging themes from the ongoing discussion among the users and their use of Facebook as their platform to promote their anti-vaccine beliefs: (1) forming an online “community” consisting of like-minded individuals and similar beliefs, (2) the widespread reach of anti-vaccine messages, and (3) debating the ethics of mandatory immunization and content moderation.

The majority of the online population of users on the Facebook Page were likely from the United States. We noticed there was a huge appreciation of community and support for one another. For example, if anyone posting antivaccine content was criticized or condemned, other users would step up to support them in the comments of posts. There was a tremendous amount of support in mother-to-mother communication. A social media analysis conducted by Gruzd and Haythornthwaite [28] analyzed a 1-month sample of Twitter messages to trace interaction via social media and understand “how a community is formed and maintained online.” The study found that network analysis can facilitate understanding of “what, and who, compromises and sustains a network…” [28]. Gruzd and Haythornwaite [28] found that active participation and attention to others were extremely significant aspects of building an online community. This finding translates very well to our analysis as aforementioned: The sharing of pictures and stories of children who were allegedly afflicted by vaccines was very popular, and mothers tended to gather and demonstrate support for one another in such cases. Supporting one another in their “time of need” and defending their collective viewpoints helped foster a sense of community among each other [29].

A very significant finding was the raising of money for private autopsies postdeath of a child from SIDS. Users believe that immunization can cause SIDS; therefore, when the cause of death is not officially linked to vaccines, users on these Facebook Pages collected donations for private autopsies, with the amounts ranging in the thousands. There was little to no follow-up from parents who received this money on whether the autopsy was done, and no follow-up included medical proof of immunization being linked to SIDS. Users on Facebook who support these Pages are a highly tight-knit community, who have limited trust in government authorities and medical professionals [30]. Users on one of the Facebook Pages examined in this research encouraged a mother to not fill a prescription for Tamiflu after her son had been diagnosed with influenza and was running a high fever and had a seizure [31]. None of the comments on the post encouraged the mother to seek medical help and fill the prescription. The mother instead opted to treat with natural remedies such as peppermint oil, vitamin C, and lavender [31]. The users on Facebook also suggested the use of home remedies such as breastmilk, thyme, and elderberry to treat her child — none of which are recommended treatment for influenza — and the child eventually died 4 days later at the hospital [31]. This case, in particular, highlights the trust and confidence users of these Pages are placing upon each other.

This relationship among users who have likely never even met each other can be incredibly difficult to infiltrate and change, as they share a common ground of strong values and beliefs. We must focus resources on individuals who are undecided and caught in the middle of the debate [11]. Interventions must be community-based, and education and information on vaccines must be encouraged by alike members in the community (such as parent groups) [32]. As health care professionals, we must become informed on adjuvants and how they impact the body (along with other concerns noted in the Results section) and therefore be equipped to answer questions parents may have to build trust [33]. It is incredibly important for health care personnel to recognize and understand this problem [33]. Vaccines are one of the largest defenses we have against communicable diseases, and we must continue to educate and attempt to change the attitudes surrounding this important issue.

### Widespread Reach of Antivaccine Messages

Antivaccine sentiments no longer belong to a small group of people; instead, they have global implications [5,11,34]. Three of the 4 Facebook Pages have 100,000 to 135,000 likes, and the most popular website shared on the Facebook Pages — Green Med Info — claims to have 500,000 monthly visitors [25]. Our research of the Pages uncovered how detrimental these campaigns can be in underdeveloped nations of the world. Pakistan is one of 3 countries that has failed to eradicate polio transmission [35]. The spread of vaccine misinformation through the availability of smartphones and social media is encouraging a public health threat in one of the most vulnerable nations of the world [34,35]. Not only has misinformation threatened Pakistan’s public health but the unauthorized immunization campaign directed by the Central Intelligence Agency in an attempt to locate Osama Bin Laden has broken the trust between locals and foreign public health efforts [36]. Another example of mistrust of western medicine in the developing world is Nigeria, where Islamic militant groups believe that immunization is a ploy to sterilize Muslims [37].

Measles outbreaks have been on the rise globally, increasing by 30% from 2016 to 2017 [38]. These outbreaks correlate heavily with whether citizens trust vaccines. For example, France experienced measles outbreaks, with 1 in 3 of their citizens believing that vaccines are not safe [38]. Currently, the COVID-19 pandemic has taken the world by storm, and researchers and the general public are eager for a vaccine to help flatten the curve of the disease. When the time comes to distribute a vaccine for this virus (or any uncontrollable communicable disease), we must not only promote the vaccine to those who trust immunization but also promote targeted interventions for users of these Pages who refuse to vaccinate by increasing opportunity for dialogue and creating safe spaces [32,33]. Our recommendation for institutions includes adding and enhancing education on vaccines and immunization for future health care professionals, such as doctors, nurses, and dentists, to address concerns of users on these Facebook Pages [33,39].

### Debating the Ethics of Mandatory Immunization and Content Moderation

A focal point of discussion on Facebook posts, website shares, and the comments section was the ethics of mandatory immunization laws [40]. Antivaccine groups are heavily against the passing of any bill that supports mandatory immunization [41]. Vaccine Choice Canada — one of Canada’s largest antivaccine organizations — claims they are prepared to fight New Brunswick’s 2019 proposed bill that will not allow children to attend public school without proof of immunization [42]. This nightmare for antivaccine organizations has already become a reality for those living in the state of California in the United States. There were calls to action against the governments that propose mandatory immunization and consistent derogatory comments made against the governor of California for being an open vaccine supporter on Facebook. Users on Facebook urged others to join protests, sign petitions, and call government officials’ offices to discourage mandatory immunization. Users frequently cited the Constitution of the United States, claiming their rights as citizens of the country have been violated through these bills and laws.

The ethical debate has also included the censoring of content on social media websites. Facebook has claimed to not remove vaccine misinformation; however, they assured the public that they will make it less prominent [7,43,44]. This includes removing content from recommended groups and ranking posts with misinformation lower on the newsfeed [7,44]. Other social media sites that are moderating antivaccine content include YouTube, Pinterest, and Twitter. YouTube has stopped serving ads on any antivaccine-promoting video and attempted to make content on the benefits of immunization easier to find [45]. The WHO has applauded Pinterest for being a leader in removing vaccine misinformation from their website [44]. Pinterest has one of the most rigorous restrictions on posting of vaccine misinformation, going as far as to block any searches with the terms “anti-vaccination” or “anti-vax” [44].

### Conclusion

The antivaccine campaign has unfortunately used social media as a vessel to spread misinformation to users, especially parents [4-7,9,11-13,26,27,29,30,32,37,40,41,46,47]. As vaccine hesitancy increases, we increase the risk of a public health crisis and lessen our chances of controlling crises like the COVID-19 pandemic. Although users on Facebook have mentioned “Truths,” the number of “Myths” supersede these truths, and the benefits of immunization greatly outweigh the risk. We must understand the local-global implications of allowing preventable diseases to make a comeback. Health care providers deal directly with members of the public who are uncertain about immunization, and it becomes their job to be informed on users’ common misconceptions [33,39]. Fake news travels faster than truth, building momentum [48]; therefore, targeted promotion of vaccines that address specific claims on the internet is warranted.

Limitations of this study include the ever-dynamic nature of the internet, with the freedom for administrators and users to remove posts as desired, and the time constraints in which we studied the Facebook Pages. The social media audit template used to organize and categorize data has previously not been used in studying data for the interpretation of messages. We also cannot be certain that each different follower of the Page is a different individual, as one person may hold many accounts using different email addresses. This analysis focuses only on Facebook, which, as aforementioned, has a less scrutinizing approach to removing antivaccine content; therefore, analysis of other websites such as Twitter, YouTube, and Pinterest is warranted to compare the type of content and spread of readership. The worldview of both authors is that of nurses and health care providers, and this article has been written with a pro-immunization point of view.

## Abbreviations

CDC: Centers for Disease Control and Prevention
MMR: measles/mumps/rubella
SIDS: sudden infant death syndrome
WHO: World Health Organization

## References

[1] (2019). Immunization. World Health Organization.

[2] (2019). Measles in Canada. Government of Canada.

[3] (2018). Poliomyelitis (Polio): For health professionals. Government of Canada.

[4] (2019). Ten threats to global health in 2019. World Health Organization.

[5] Benecke O, DeYoung SE (2019). Anti-Vaccine Decision-Making and Measles Resurgence in the United States. Glob Pediatr Health.

[6] Stahl J, Cohen R, Denis F, Gaudelus J, Martinot A, Lery T, Lepetit H (2016). The impact of the web and social networks on vaccination. New challenges and opportunities offered to fight against vaccine hesitancy. Med Mal Infect.

[7] Caron C (2019). Facebook Announces Plan to Curb Vaccine Misinformation. The New York Times.

[8] Clement J (2020). Most popular social networks worldwide as of July 2020, ranked by number of active users. Statista.

[9] Hussain A, Ali S, Ahmed M, Hussain S (2018). The Anti-vaccination Movement: A Regression in Modern Medicine. Cureus.

[10] Martin B (2020). Texas Anti-Vaxxers Fear Mandatory COVID-19 Vaccines More Than the Virus Itself. TexasMonthly.

[11] Johnson N, Velásquez N, Restrepo N, Leahy R, Gabriel N, El Oud S, Zheng M, Manrique P, Wuchty S, Lupu Y (2020). The online competition between pro- and anti-vaccination views. Nature.

[12] (2020). WHO fights a pandemic besides coronavirus: An "infodemic". The New York Times.

[13] (2020). Surge of virus misinformation stumps Facebook and Twitter. The New York Times.

[14] (2019). Frequently Asked Flu Questions 2018-2019 Influenza Season. Centers for Disease Control and Prevention.

[15] Quesenberry K (2017). Social media not meeting expectations? Perform a social media audit.

[16] Quesenberry KA (2015). Conducting a Social Media Audit. Harvard Business Review.

[17] Centers for Disease Control and Prevention (2020). Measles cases in 2019. Measles Cases and Outbreaks.

[18] Rodriguez NJ (2016). Vaccine-Hesitant Justifications: "Too Many, Too Soon," Narrative Persuasion, and the Conflation of Expertise. Glob Qual Nurs Res.

[19] Christensen D (2016). Vaccine adjuvants: Why and how. Hum Vaccin Immunother.

[20] (2018). Selecting viruses for the seasonal influenza vaccine. Centers for Disease Control and Prevention.

[21] (2019). Flu (influenza): Get your flu shot. Government of Canada.

[22] Schwartz KL, Kwong JC, Deeks SL, Campitelli MA, Jamieson FB, Marchand-Austin A, Stukel TA, Rosella L, Daneman N, Bolotin S, Drews SJ, Rilkoff H, Crowcroft NS (2016). Effectiveness of pertussis vaccination and duration of immunity. CMAJ.

[23] Klein NP, Bartlett J, Fireman B, Rowhani-Rahbar A, Baxter R (2013). Comparative effectiveness of acellular versus whole-cell pertussis vaccines in teenagers. Pediatrics.

[24] Funk S (2017). Critical immunity thresholds for measles elimination.

[25] (2020). GreenMedinfo.

[26] Ward JK, Peretti-Watel P, Verger P (2016). Vaccine criticism on the Internet: Propositions for future research. Hum Vaccin Immunother.

[27] Smith TC (2017). Vaccine Rejection and Hesitancy: A Review and Call to Action. Open Forum Infect Dis.

[28] Gruzd A, Haythornthwaite C (2013). Enabling community through social media. J Med Internet Res.

[29] Vrdelja M, Kraigher A, Vercic D, Kropivnik S (2018). The growing vaccine hesitancy: exploring the influence of the internet. Eur J Public Health.

[30] Orr D, Baram-Tsabari A, Landsman K (2016). Social media as a platform for health-related public debates and discussions: the Polio vaccine on Facebook. Isr J Health Policy Res.

[31] Zadrozny B (2020). On Facebook, anti-vaxxers urged a mom not to give her son Tamiflu. He later died. NBC News.

[32] Steffens M, Dunn AG, Wiley KE, Leask J (2019). How organisations promoting vaccination respond to misinformation on social media: a qualitative investigation. BMC Public Health.

[33] Paterson P, Meurice F, Stanberry LR, Glismann S, Rosenthal SL, Larson HJ (2016). Vaccine hesitancy and healthcare providers. Vaccine.

[34] Dubé E, Gagnon D, Nickels E, Jeram S, Schuster M (2014). Mapping vaccine hesitancy--country-specific characteristics of a global phenomenon. Vaccine.

[35] Saifi S, Shah S (2019). Pakistan's anti-vaccination movement leads to string of deadly attacks. CNN.

[36] (2015). He led the CIA to bin Laden- and unwittingly fueled a vaccine backlash.

[37] (2019). Vaccines: Low trust in vaccination 'a global crisis'. BBC News.

[38] (2019). How anti-vaccine movements threaten global health. BBC News.

[39] Esposito S, Principi N, Cornaglia G, ESCMID Vaccine Study Group (EVASG) (2014). Barriers to the vaccination of children and adolescents and possible solutions. Clin Microbiol Infect.

[40] Williamson L, Glaab H (2018). Addressing vaccine hesitancy requires an ethically consistent health strategy. BMC Med Ethics.

[41] Whelan AM (2016). Lowering the Age of Consent: Pushing Back against the Anti-Vaccine Movement. J Law Med Ethics.

[42] Brown S (2019). Anti-vaccination group to challenge New Brunswick mandatory immunization. Global News.

[43] Pagoto S, Waring ME, Xu R (2019). A Call for a Public Health Agenda for Social Media Research. J Med Internet Res.

[44] Cohen E, Bonifield J (2019). Facebook to get tougher on anti-vaxers. CNN.

[45] (2019). YouTube takes ads off 'anti-vax' video channels. BBC News.

[46] Burki T (2019). Vaccine misinformation and social media. The Lancet Digital Health.

[47] Kata A (2010). A postmodern Pandora's box: anti-vaccination misinformation on the Internet. Vaccine.

[48] Shu K, Sliva A, Wang S, Tang J, Liu H (2017). Fake News Detection on Social Media. SIGKDD Explor. Newsl.

